# Evaluating treatment options for symptomatic uterine fibroids: a systematic review and meta-analysis of effectiveness, recovery, and long-term outcomes (MARIE WP1)

**DOI:** 10.3389/fgwh.2025.1601341

**Published:** 2025-07-31

**Authors:** Elena Bedggood, Sun Jie, Snehal Ghosh, Vindya Pathiraja, Tharanga Mudalige, Nirmala Rathnayake, Heitor Cavalini, Om Kurmi, George Uchenna Eleje, Peter Phiri, Paula Briggs, Jian Qing Shi, Gayathri Delanerolle, Sohier Elneil

**Affiliations:** ^1^ Department of Applied Science, University of Birmingham, Birmingham, United Kingdom; ^2^ Department of Statistics, Southern University of Science and Technology, Shenzhen, China; ^3^ Faculty of Allied Health Science, University of Ruhuna, Matara, Sri Lanka; ^4^ Hampshire and Isle of Wight Healthcare NHS Foundation Trust, Southampton, United Kingdom; ^5^ Faculty of Health and Life Science, Coventry University, Coventry, United Kingdom; ^6^ Department of Gynaecology, Nnamdi Azikiwe University, Awka, Nigeria; ^7^ Department of Medicine, University of Southampton, Southampton, United Kingdom; ^8^ Department of Gynaecology, Liverpool Womens Hospital NHS Foundation Trust, Liverpool, United Kingdom; ^9^ Institute of Womens Health, University College London, London, United Kingdom

**Keywords:** treatment options, uterine fibroids, systematic review, meta-analysis, effectiveness

## Abstract

**Background:**

Uterine fibroids can significantly impair the quality of life of women. While most fibroids remain asymptomatic, 25% of women diagnosed with uterine fibroids require medical intervention.

**Methods:**

A systematic review and meta-analysis protocol was developed and published in PROSPERO (CRD42022346251) to explore surgical treatment outcomes linked to uterine fibroids. Data was gathered using PubMed, Web of Science and ScienceDirect. The pooled data was analysed using the meta-package (version 8.0–1 and version 4.6–0) in R software (version 4.4.2).

**Results:**

Five studies met the eligibility criteria, and were further analysed to report quality of life, symptom severity and complications linked to surgery. Three studies (*n* = 520) assessed HRQoL via UFS-QoL pre- and post-uterine artery embolisation and myomectomy. The pooled mean difference was −6.99 [95% CI: (−16.49, 2.51); *I*^2^ = 71.9%; *P* = 0.03], indicating no significant difference in quality of life impact between procedures. However, the pooled mean difference for UFS-QoL symptom severity was 4.85 [95% CI: (0.50, 9.21); *I*^2^ = 0.0%; *P* = 0.52], suggesting myomectomy significantly reduces symptom severity compared to uterine artery embolisation. Most studies did not report race and ethnicity, and the study sample was not representative of the global female populous.

**Conclusion:**

Uterine artery embolisation and myomectomy result in comparable improvements in health-related quality of life although myomectomy appears to offer a greater reduction in symptom severity compared to uterine artery embolisation. These findings can assist clinicians and patients make improved shared decisions when selecting the most appropriate treatment for uterine fibroids. Improved research study designs and representation in sample need to be considered when conducting future research.

## Introduction

Uterine fibroids (1), also known as uterine leiomyomas, are common benign tumours of the uterus that disproportionately affect women of reproductive age. While non-malignant, fibroids can significantly impair quality of life, causing symptoms such as heavy menstrual bleeding, pelvic pain, and infertility. The prevalence of UF increases with age, affecting approximately 40% of premenopausal women and nearly 50% of women by the age of 50 years (2). While most fibroids are asymptomatic, around 25% of women who develop fibroids experience symptoms severe enough to require treatment (3). These symptoms, including menorrhagia, pelvic pressure, and iron deficiency anaemia, can significantly impact physical and emotional well-being (4, 5).

The prevalence of uterine fibroids is particularly high among women of African descent, with studies indicating that 60%–80% of African American women will develop fibroids by age 50, compared to 30%–40% of White women (6). Black women also face a greater risk of requiring surgical intervention, with a 2.4-fold increased likelihood of undergoing a hysterectomy and a 6.8-fold higher chance of requiring multiple myomectomies (7). These racial disparities reflect both biological factors and disparities in healthcare access, as women in lower-income regions or with limited access to care face greater challenges in managing the condition (5).

In high-income countries, fibroid treatment benefits from advanced healthcare systems, where access to diagnostic tools such as high-resolution ultrasound and magnetic resonance imaging (MRI) enables early detection and personalized care. Treatment options range from pharmacological therapies, such as hormonal treatments (gonadotropin-releasing hormone (GnRH) agonists, oral contraceptives, and progesterone-releasing intrauterine devices (IUDs), to minimally invasive procedures like uterine artery embolization [UAE] (8), MRI-guided focused ultrasound, and robotic-assisted myomectomy (6, 9). For definitive management, hysterectomy remains a common option, with robotic and laparoscopic approaches offering quicker recovery times and fewer complications. These treatments are supported by comprehensive insurance coverage and a well-developed specialist workforce, ensuring women have access to timely and effective care (10–12).

However, in low- and middle-income countries (LMICs), fibroids management is constrained by limited healthcare resources, systemic challenges, and geographical disparities. Diagnostic capabilities are often limited to basic ultrasound, with advanced imaging modalities largely unavailable in rural or underserved regions. Medical treatments, including hormonal therapies, are hindered by inconsistent access to affordable medications and poor pharmaceutical supply chains. Advanced treatments like UAE and minimally invasive surgery are mostly confined to urban centres or private healthcare facilities, leaving many women with limited access to these options (5, 7). In such settings, surgical interventions often include open myomectomy or hysterectomy, which may carry higher risks due to inadequate perioperative care (12, 13).

Cultural factors and financial constraints in LMICs can also lead women to seek traditional remedies or community-based care. While these approaches may be culturally significant, they often lack the evidence base and effectiveness of modern medical treatments (14). This reliance on traditional practices further highlights the healthcare disparities between resource-rich and resource-constrained settings, where access to optimal care for fibroids remains limited (15, 16).

The global management of fibroids reflects stark disparities between developed countries and LMICs. In high-income nations, women have access to a range of advanced diagnostic and treatment options, ensuring timely and effective management. In contrast, women in LMICs often face significant barriers to care, with fewer treatment options and limited access to healthcare infrastructure. Currently, no published meta-analysis has examined the impact on postoperative quality of life specifically for UAE, hysterectomy, and myomectomy. This article compares the impact of UAE, myomectomy, and hysterectomy on women's quality of life, assess changes in symptom severity, and examine the occurrence of complications such as haemorrhage, urinary retention, infection, and vaginal discharge following each treatment of uterine fibroids (17). The study also examines the prevalence and treatment landscape of fibroids worldwide, highlighting the significant disparities in access to care, and proposes strategies to bridge these gaps. By fostering equitable healthcare solutions and increasing access to uterine-preserving treatments, we can improve outcomes for women with fibroids, regardless of their geographic location.

## Methods

A systematic methodology was developed and published in PROSPERO (CRD42022346251) to explore outcomes based on surgical treatments among patients with uterine fibroids.

### Search strategy and screening

A search was conducted using PubMed, Web of Science and Science Direct. The key terms used were *uterine fibroids and* surgical treatment outcomes.

### Eligibility criteria and study selection

All studies that were included into the study sample comprised of quantifiable measures and outcomes linked to uterine fibroids. All randomised clinical trials, non-randomised clinical trials, mixed-methods, and epidemiology studies that reported on uterine fibroids that were peer reviewed and published in English from the 30th of April 1980 to the 30th of September 2024 were included to the data sample.

### Data extraction and analysis

**Figure 1:**
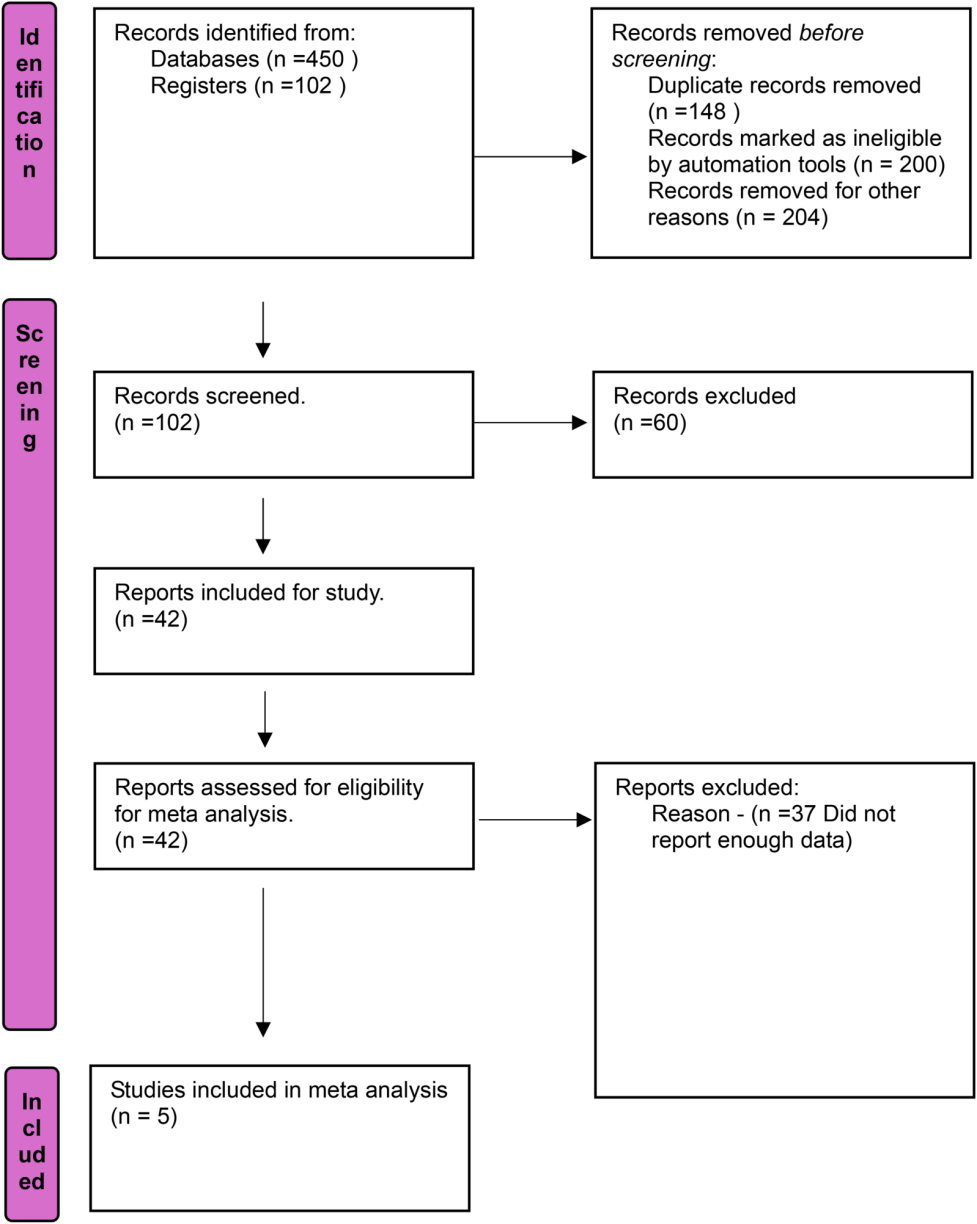
PRISMA flow diagram.

The data extraction process is demonstrated in the PRISMA diagram (Figure 1). All quantifiable measures of mean, median, mode, standard deviation, were extracted from all the studies. The data was refined using an independent reviewer and pooled using Endnote and Microsoft Excel by 2 reviewers (EB and JS). Finally, 42 articles were assessed for eligibility (Table 1). Pooled odds ratios (18) and 95% confidence intervals (19) were reported. Also, the sample sizes reported in Table 1 adhere to the intention-to-treat (ITT) principle, reflecting the number of participants randomized in the study. This approach also ensures consistency within Table 1, as different outcomes in one study often had varying sample sizes. For each individual analysis, the sample size incorporated was that specifically reported for the relevant outcome in each study.

**Table 1:** Characteristics of the studies included in the systematic review.

ID	Author	Country	Study type	Participants	Intervention (Exposure)	Control	Sample Size	Outcomes	Included (Y/N)
1	Manyonda et al. (20)	UK	RCT	Women with uterine fibroids	UAE	Hysterectomy	127/127	UFS-QoL, EuroQol-5	Y
2	Moss et al. (21)	UK	RCT	Women with uterine fibroid	UAE	Hysterectomy or Myomectomy	106/51	SF-36 QoL	N
3	Don et al. (2)	Netherland	Cohort	Women with uterine fibroid	Myomectomy	Expectant management	165/146	Time to live birth	N
4	Goodwin et al. (8)	USA	Cohort	Women with uterine fibroid	UAE	Myomectomy	149/60	QoL Menstrual bleeding, Uterine size and Adverse events.	Y
5	Homer et al. (22)	-	Review	Women with uterine fibroid	UAE	-	227	Miscarriage, Preterm delivery, malpresentation, Intrauterine growth restriction (IUGR), Cesarean delivery, and Postpartum hemorrhage (PPH)	N
6	Lethaby et al. (23)	-	Review	Premenopausal women with fibroids	Gonadotrophin releasing hormone (GnRH) analogues (GnRHa)	No treatment, Placebo or other Medical treatment.	-	Pre-operative. Intra-operative, Post-operative assessment	N
7	Chittawar et al. (6)	-	Review	Women with uterine fibroid	Laparoscopic or hysteroscopic myomectomy	Open myomectomy	-	Postoperative pain, In-hospital adverse events	N
8	de Bruijn et al. (13)	Netherland	RCT	Women with uterine fibroid	UAE	Hysterectomy	81/75	Additional interventions HRQOL, Urinary and defecation function, Menopausal symptoms, Menstrual characteristics	Y
9	Hehenkamp et al. (14)	Netherland	RCT	Women with uterine fibroid	UAE	Hysterectomy	88/89	Sexual Activity Questionnaire [SAQ] and the Body Image Scale [BIS]	N
10	Mara et al. (24)	Czech	case-series	UAE performed women	UAE	-	51	Hysteroscopy and endometrial biopsy	N
11	Panagiotopoulou et al. (25)	-	Review	Women with uterine fibroid	UAE	Laparoscopic uterine artery occlusion, Myomectomy	436	Patient satisfaction, Clinical failure	N
12	Daniels et al. (26)	UK	RCT	Premenopausal women with uterine fibroids	UAE	Myomectomy	127/127	UFS-QoL, EuroQol-5	N
13	Manyonda et al. (18)	UK	RCT	Women with uterine fibroid	UAE	Myomectomy	81/81	UFS-QOL, Complications	Y
14	Edwards et al. (27)	UK	RCT	Women with uterine fibroid	UAE	Hysterectomy or Myomectomy	106/51	SF-36 QoL	Y
15	Jun et al. (10)	China	RCT & Review	Women with uterine fibroid	UAE	Surgery	63/64	QoL, Hospital stay	N
16	Hehenkamp et al. (28)	Netherland	RCT	Pre-menopausal women with menorrhagia	UAE	Hysterectomy	88/89	Follicle stimulating hormone (FSH) and anti-Mullerian hormone (AMH).	N
17	Ruuskanen et al. (4)	Finland	RCT	Women with uterine fibroid	UAE	Hysterectomy	27/30	Improvement of symptoms.	N
18	Mara et al. (29)	Czech	RCT	women with intramural fibroid(s) larger than 4 cm	UAE	Myomectomy	30/33	Invasiveness, efficacy, and complications	N
19	Laughlin-Tommaso et al. (11)	USA	RCT	Premenopausal women with symptomatic uterine fibroids	UAE	Magnetic resonance imaging–guided focused ultrasound surgery	40/43	Reintervention for uterine fibroids, Serum anti-Müllerian hormone levels	N
20	Laughlin-Tommaso et al. (30)	USA	RCT	Women with uterine fibroid	UAE	Magnetic resonance imaging-guided focused ultrasound surgery (MRgFUS)	7/8	Fibroid load reduction,Volume of the largest fibroid	N
21	AbdElmagied et al. (31)	USA	RCT	Women with uterine fibroid	UAE	Magnetic resonance imaging-guided focused ultrasound surgery (MRgFUS)	18/16	McGill Pain Score,Visual Analog Scale scores	N
22	Bray et al. (32)	USA	Cross-sectional	African American (AA) participants with image- or surgery-confirmed fibroids	African ancestry	European ancestry	609	Fibroid number, volume of largest fibroid, and largest fibroid dimension of all fibroid measurements.	N
23	Shen et al. (33)	China	RCT	Women with uterine fibroid	UAE using Embosphere microspheres	UAE using panhysterectomy	64/64	Serum TGF-b levels	N
24	Pritts et al. (34)	-	Review	Women with fibroids and infertility.	Women with fibroids	Women without fibroids	-	Clinical pregnancy rate, spontaneous abortion rate, ongoing pregnancy/live birth rate	N
25	Surrey et al. (35)	USA	Case-Control	Consecutive fresh IVF-ET and oocyte donation patients	Precycle hysteroscopic or abdominal myomectomy and subsequent fresh IVF-ET	Oocyte donation.	46/55	Vitro fertilization-embryo transfer (IVF-ET) and oocyte donation cycle	N
26	Firouznia et al. (36)	Iran	Case-series	Women with uterine fibroid	UAE	-	15	Pregnancies and outcome	N
27	Berman et al. (37)	USA	Cohort	Women with uterine fibroid	Black women	White women	28/46	QoL, Symptom severity score (SSS)	N
28	Stewart et al. (3)	Multination	RCT	Women with uterine fibroid	Relugolix combination therapy	Placebo	253/256	QoL, emotional well-being, physical and social, activities, and sexual function	N
29	Styer et al. (38)	USA	RCT	Couples with unexplained infertility	Clomiphene citrate, letrozole,	Gonadotropins	300/299/301	Live birth rate	N
30	Stewart et al. (39)	USA	RCT	Premenopausal women with HMB associated with uterine fibroids	Oral asoprisnil 10 mg, asoprisnil 25 mg	Placebo	317/321/153	Menstrual bleeding Volume of the largest fibroids, Uterine volume and HRQoL	N
31	Sener et al. (40)	Turkey	RCT	Postmenopausal women with small asymptomatic uterine fibroids	50 f1 g transdermal E2 plus 5 mg medroxyprogesterone acetate (MPA)	0.625 mg conjugated equine estrogen plus 2.5 mg MPA	40	The size of the uterine fibroids	N
32	Stewart et al. (5)	USA	RCT	African American premenopausal women (aged 18e50 years) with uterine fibroids and heavy menstrual bleeding	Open-label relugolix combination therapy	Placebo	89/82/70	Uerine fibroid-associated heavy menstrual bleeding	N
33	Wise et al. (41)	USA	Cohort	African American women	Intact uteri and no prior diagnosis of uterine leiomyomata	-	21,506	Risk of uterine leiomyomata	N
34	Shen et al. (42)	China	Cohort	Asian women	Patients with uterine leiomyoma	-	21,168	The risk of depression	N
35	Mitro et al. (43)	USA	Cohort	Pregnant women	racially diverse	-	2,774	Fibroid number and volume	N
36	Huyck et al. (12)	USA	Case-control	Family history of UL	Black women	White women	285	Severity of UL	N
37	Maleux et al. (9)	Belgium	RCT	Women with uterine fibroid	3D roadmap technique UAE	Conventional two-dimensional (2D) roadmap UAE	40	Ovarian dose, total contrast load	N
38	Wu et al. (44)	USA	Cross-sectional	Women aged 16 years or older who underwent a hysterectomy	Hysterectomy types	-	602,457	Hysterectomy rates	N
39	Hendy et al. (45)	Multination	RCT	Women with fibroid-associated heavy menstrual bleeding	Relugolix combination therapy or Delayed relugolix combination therapy	Placebo	132/128/127	Amenorrhea, volume of menstrual blood loss, distress from bleeding and pelvic discomfort, anemia, pain, fibroid volume, and uterine volum	N
40	Li et al. (46)	-	Review	Women with uterine fibroid	-	-	237,509	Risks of adverse pregnancy and obstetric	N
41	Donnez et al. (47)	Multination	RCT	Women with symptomatic uterine fibroids before surgery	Oral ulipristal acetate(5 mg/10 mg)	Placebo	96/98/48	Uterine bleeding	N
42	Schlaff et al. (48)	USA	RCT	Women with fibroid-associated bleeding	Elagolix Alone/Elagolix with Add-Back Therapy	Placebo	104/206/104	Menstrual blood loss	N

### Statistical analysis

Since both myomectomy and hysterectomy are primary surgical interventions for symptomatic uterine fibroids (leiomyomas) and other gynecological conditions—and since the outcomes of interest (e.g., quality of life) are structurally comparable—we combined myomectomy and hysterectomy into a single surgical group for comparison with uterine artery embolization (8). This approach aligns with Study 14 and multiple published systematic reviews that have combined myomectomy and hysterectomy data.

The primary outcome was postoperative quality of life in patients undergoing UAE, myomectomy, or hysterectomy, assessed via validated scales such as: UFS-QOL, EQ-5D-3l, and SF-36. For each study, we extracted or calculated the mean change from baseline to postoperative follow-up (with standard deviations) in QoL scores. We then computed the difference in mean change between the UAE group and the combined surgical group (myomectomy + hysterectomy) and pooled these results across studies in the meta-analysis. For complications, we calculated the incidence rates of post-embolization syndrome, urinary retention, infection, and vaginal discharge for each study and derived overall estimates. Additionally, we extracted the number of hemorrhage events and person-time data for both groups, computed odds ratios (OR) with 95% confidence intervals (CI), and performed meta-analysis to obtain pooled estimates.

All outcome data were either directly extracted from study reports or calculated using methods recommended by the Cochrane Handbook (49). Outcome data reported at the 1-year follow-up were used for analysis; if 1-year follow-up was unavailable, the longest available follow-up (e.g., 6 months) was used instead.

All meta-analyses used either random-effects models or fixed-effects models based on the heterogeneity between studies. Statistical heterogeneity among studies was assessed using the Cochrane *Q*-test and the *I*^2^ statistic. Random-effects models were implemented when the Cochrane *Q*-test yielded *p* < 0.05 or *I*^2^ > 50%; otherwise, fixed-effects models were applied. In cases of significant heterogeneity, we planned to perform subgroup analyses and meta-regression to explore sources of heterogeneity. We also intended to use Egger's test to assess potential publication bias. However, these analyses were not conducted because each meta-analysis included fewer than five studies. With such a limited number of studies, both subgroup analyses/meta-regression and Egger's test lack sufficient statistical power and may yield misleading results. Therefore, these methods were not applied. Therefore, we implemented narrative synthesis to assess publication bias. Data analysis was performed using the meta package (version 8.0-1) and the metafor package (version 4.6-0) in R software (version 4.4.2).

### Risk of bias

The Newcastle Ottawa Quality Assessment Scale (8) was used to assess the risk of bias. The studies were evaluated using the following criteria: selection, comparability and exposure. A maximum of four stars was awarded for selection, two for comparability and three for outcomes, with a maximum of nine stars. NOS was used to assess the quality of cohort studies, case control and cross-sectional studies. The studies were categorised into low risk if they scored 7–9 stars, moderate risk if they scored 5–6 stars and high risk if they scored 0–4 stars (Table 2).

**Table 2:** Risk of bias for case control/cohort and cross-sectional studies.

No	Authors	Selection (S)				Comparability (C)	Exposure/Outcome (E/O)			Total Stars	Conclusion
		1	2	3	4	1	1	2	3		
1	Don, E. E. et al.	*	*	*	*	**	*	*	*	*********	Low risk
2	Goodwin, S. C et al.	*	*	*	*	**	*	*	*	*********	Low risk
3	Bray, M. J. et al.	*	*	*	*	**		*	*	********	Low risk
4	Surrey, E. et al.	*	*	*	*	**	*	*	*	*********	Low risk
5	Firouznia, K. et al.	*	*	*	*	**	*	*	*	*********	Low risk
6	Wise, L. A. et al.	*	*	*	*	**	*	*	*	*********	Low risk
7	Shen, T. C. et al.	*	*	*	*	**	*	*	*	*********	Low risk
8	Mitro, S. D. et al.	*	*	*	*	**	*	*	*	*********	Low risk
9	Huyck, K. L. et al.	*		*	*	**	*	*	*	********	Low risk
10	Wu, J. et al.	*	*		*	*	*	*		******	Moderate risk

And Cochrane Collaboration's tool was used to assess the risk of bias in randomized control trials (Table 3). The studies were catergorised into low risk of bias (The trial is judged to be at low risk of bias for all domains for this result), some concerns (The trial is judged to raise some concerns in at least one domain for this result, but not to be at high risk of bias for any domain) and high risk of bias (The trial is judged to be at high risk of bias in at least one domain for this result or The trial is judged to have some concerns for multiple domains in a way that substantially lowers confidence in the result) accordingly.

**Table 3:** Risk of bias for randomized control trials using cochrane collaboration's tool.

No.	Authors	Randomization Process	Deviation from the intended interventions	Missing outcome data	Measurement of outcome	Selection of the reported results	Overall
1.	Manyonda, I. et al.	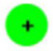	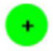	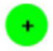	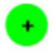	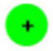	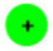
2.	Moss, J. G. et al.	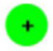	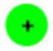	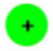	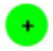	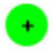	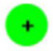
3.	de Bruijn, A. M. et al.	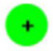	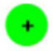	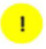	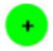	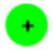	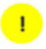
4.	Hehenkamp, W. J. et al.	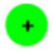	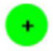	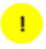	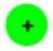	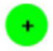	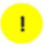
5.	Daniels, J. et al.	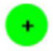	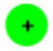	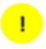	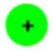	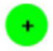	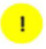
6.	Manyonda, I. et al.	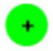	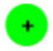	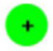	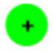	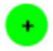	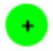
7.	Edwards, R. et al.	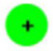	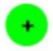	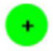	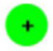	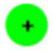	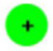
8.	Jun, F. et al.	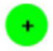	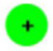	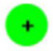	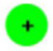	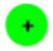	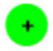
9.	Hehenkamp, W. et al.	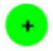	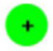	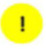	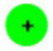	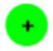	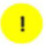
10.	Ruuskanen, A. et al	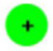	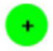	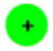	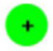	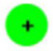	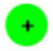
11.	Mara, M. et al.	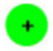	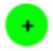	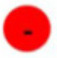	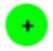	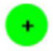	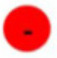
12.	Laughlin-Tommaso, S. et al.(2018)	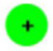	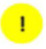	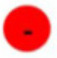	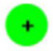	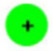	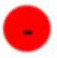
13.	Laughlin-Tommaso, S. K. et al. (2022)	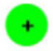	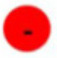	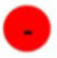	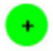	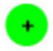	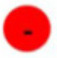
14.	AbdElmagied, A. M. et al.	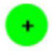	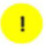	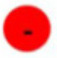	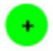	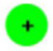	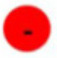
15.	Berman, J. M. et al.	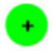	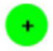	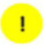	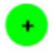	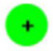	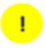
16.	Stewart, E. A. et al. (2023)	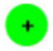	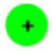	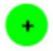	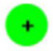	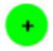	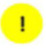
17.	Styer, A. et al	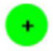	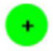	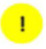	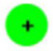	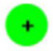	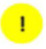
18.	Stewart, E. A. et al. (2019)	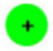	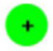	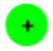	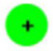	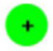	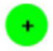
19.	Sener, A. B. et al.	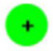	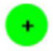	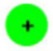	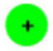	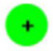	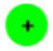
20.	Stewart, E. A. et al.(2024)	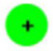	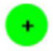	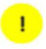	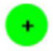	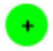	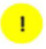
21.	Maleux, G. et al.	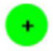	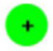	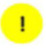	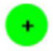	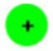	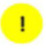
22.	Al-Hendy, A. et al.	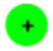	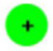	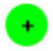	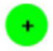	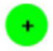	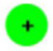
23.	Donnez, J. et al.	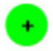	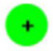	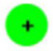	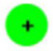	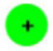	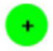
24.	Schlaff, W. D. et al.	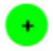	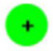	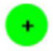	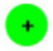	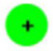	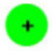
25.	Shen, T. et al.	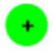	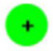	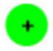	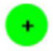	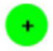	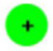

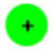
 Low risk.

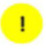
 Some Concerns.

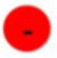
 High risk.

## Results

### Quality of life

Uterine fibroids can significantly impair quality of life. For women who do not respond to medical treatment, there are typically three treatment options: UAE, myomectomy, and hysterectomy. We will conduct a meta-analysis to compare the impact of UAE with myomectomy and hysterectomy on women's quality of life. Additionally, the characteristics of participants from the five studies included in the meta-analysis are presented in Table 4.

**Table 4:** Characteristics of patients of the included studies.

No.	Authors	Publication Year	Groups	Age of the UAE Group (Mean ± SD)	Age of the Surgical Group (Mean ± SD)	Uterine Volume of the UAE Group—cm^3^	Uterine Volume of the Surgical Group—cm^3^	Largest fibroid volume of the UAE Group—cm^3^	Largest fibroid volume of the Surgical Group—cm3	Largest fibroid diameter of the UAE Group—cm	Largest fibroid diameter of the Surgical Group—cm	Time points for outcome measurement
1	Manyonda, I. et al.	2020	UAE vs. Myomectomy	40.2 ± 6.55	42.7 ± 6.4	1170 ± 1,280 (Mean ± SD)	1,240 ± 1,120 (Mean ± SD)	436 ± 594 (Mean ± SD)	446 ± 548 (Mean ± SD)	-	-	Baseline, 6 months, 1years, 2 years
4	Goodwin et al.	2006	UAE vs. Myomectomy	-	-	658.4 (Mean)	590.6 (Mean)	182.12 ± 208.978 (Mean ± SD)	226.92 ± 196.394 (Mean ± SD)	-	-	3 months, 6 months
8	de Bruijn et al.	2016	UAE vs. Hysterectomy	44.6 ± 4.8	45.4 ± 4.2	321 (31-3,005) Median (range)	313 (58-3,617) Median (range)	59 (1-673) Median (range)	87 (4-1,641) Median (range)	-	-	1 year, 2 years, 5 years, 10 years
13	Manyonda et al.	2012	UAE vs. Myomectomy	44 ± 5.7	43.2 ± 5.3	973 ± 946.8 (Mean ± SD)	707.1 ± 511.8 (Mean ± SD)	-	-	7.7 ± 3.8 (Mean ± SD)	6.53 ± 2.8 (Mean ± SD)	1 month. 1 year

**Table 5:** List ost-operative complications.

Post-operative complication	Description
Haemorrhage	Haemorrhage is a common and potentially serious postoperative complication in patients undergoing surgery for uterine fibroids, particularly myomectomy. The vascular nature of fibroids and extensive dissection required can lead to significant intraoperative or postoperative bleeding. Risk factors include large fibroid size, multiple fibroids, and prior uterine surgery. Excessive bleeding may necessitate blood transfusion, surgical re-exploration, or conversion to hysterectomy. Preventative strategies include preoperative optimisation such as the use of GnRH analogues, meticulous surgical technique, and intraoperative use of vasoconstrictive agents. Prompt recognition and management are essential to prevent hypovolaemic shock, prolonged hospital stay, and adverse reproductive outcomes
Post emobolisation syndrome	Post-embolisation syndrome (PES) is a frequent complication following uterine artery embolisation for fibroid treatment. It typically occurs within 24–72 h post-procedure and is characterised by pelvic pain, low-grade fever, nausea, fatigue, and leukocytosis. PES results from ischaemic necrosis of fibroid tissue, triggering an inflammatory response. Although self-limiting, it can cause significant discomfort and prolong hospitalisation. Management is supportive, involving analgesics, antipyretics, hydration, and antiemetics. Differentiation from serious complications such as infection or sepsis is crucial. Educating patients about PES prior to UAE is important to reduce anxiety and ensure timely reporting of symptoms for appropriate care
Urinary retention	Urinary retention is an occasional postoperative complication following uterine fibroid surgery, particularly after abdominal myomectomy or hysterectomy. It may result from pain-induced reflex inhibition, perioperative use of opioids, or injury to the pelvic nerves during dissection. Bladder overdistension, catheter-associated discomfort, or pelvic haematoma compressing the bladder outlet may also contribute. Patients may present with lower abdominal pain, discomfort, or an inability to void. Diagnosis is confirmed via bladder scanning or catheterisation. Management includes temporary catheterisation, analgesia, and monitoring of urinary function. Persistent retention may necessitate urological review. Early mobilisation and careful intraoperative technique reduce the risk of this complication
Infection	Infection is a notable postoperative complication following uterine fibroid surgery, with risk influenced by surgical route, procedure duration, and patient comorbidities. Common infections include wound infections, urinary tract infections, and pelvic abscesses. Open myomectomy and hysterectomy carry higher risks compared to minimally invasive techniques. Clinical features may include fever, localised pain, erythema, discharge, or systemic signs of sepsis. Prophylactic antibiotics, aseptic technique, and early ambulation are essential preventative strategies. Infections are typically managed with antibiotics, though abscesses may require drainage. Prompt recognition and treatment are vital to prevent prolonged recovery, hospital readmission, and negative reproductive or general health outcomes
Vaginal discharge	Vaginal discharge is a common postoperative symptom, particularly following uterine fibroid procedures involving the uterine cavity, such as hysteroscopic myomectomy or uterine artery embolisation. It may result from sloughing of necrotic fibroid tissue, ischaemia-induced endometrial shedding, or low-grade inflammation. While often benign and self-limiting, discharge may persist for several weeks and can be watery, blood-stained, or mildly odorous. However, foul-smelling or purulent discharge warrants evaluation for infection or retained tissue. Patient education is crucial to distinguish normal postoperative changes from pathological signs. If necessary, treatment includes antibiotics and imaging to exclude complications such as infection or fistula formation

### The uterine fibroid symptom and health-related quality of life questionnaire (UFS-QOL)

The Uterine Fibroid Symptom and Health-Related Quality of Life Questionnaire (UFS-QOL) is a scale specifically designed to assess the quality of life in patients with uterine fibroids. This scale is divided into two main sections: health-related quality of life and symptom severity. In the following analysis, we will compare the changes in patients' quality of life before and after UAE and myomectomy.

### UFS-QOL health-related quality-of-life

**Figure 2:**
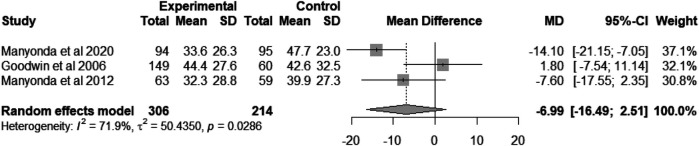
Forest plot showing the mean difference of UFS-QoL health-related quality-of-life score between UAE group and myomectomy group.

A total of three studies involving 520 patients reported changes in health-related quality of life (HRQoL) as measured by the UFS-QoL scale before and after undergoing UAE and myomectomy. The pooled mean difference was −6.99 [95% CI = (−16.49, 2.51), heterogeneity *I*^2^ = 71.9%, *P* = 0.03; Figure 2] (8, 18, 20). According to the 95% confidence interval, there is no statistically significant difference in the impact on patients' quality of life between UAE and myomectomy.

### UFS-QOL symptom severity

**Figure 3:**
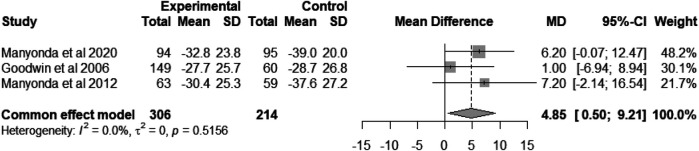
Forest plot showing the mean difference of UFS-QoL symptom severity score between UAE group and myomectomy group.

The pooled mean difference for the UFS-QoL symptom severity was 4.85 [95% CI = (0.50, 9.21), heterogeneity *I*^2^ = 0.0%, *P* = 0.52; Figure 3] (8, 18, 20). This indicates that while there is no difference between the two procedures in improving patients' quality of life, myomectomy significantly reduces symptom severity compared to UAE.

### European quality of life 5 dimensions 3 Level version(EQ-5D-3l)

**Figure 4:**
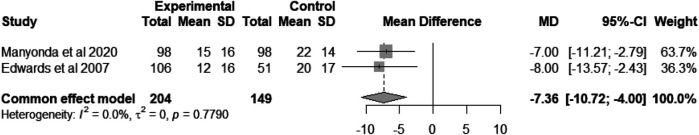
Forest plot showing the mean difference of EQ-5D-3l score between UAE group and myomectomy or hysterectomy group.

Some studies have also used the EQ-5D-3l scale to measure patients' quality of life. The following analysis compares the changes in EQ-5D-3l quality of life scores before and after treatment in patients undergoing UAE vs. those undergoing myomectomy or hysterectomy. According to reports from two studies involving a total of 353 patients, the pooled mean difference was −7.36 [95% CI = (−10.72, −4.00), heterogeneity *I*^2^ = 0.0%, *P* = 0.78; Figure 4] (20, 27). This indicates that, when using the EQ-5D-3l scale to measure quality of life, the myomectomy or hysterectomy group showed a statistically significant improvement in quality of life compared to the UAE (8) group.

### The 36-item short form health survey (SF-36)

**Figure 5:**
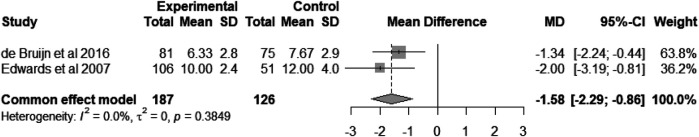
Forest plot showing the mean difference of SF-36(mental component summary) between UAE group and myomectomy or hysterectomy group.

**Figure 6:**
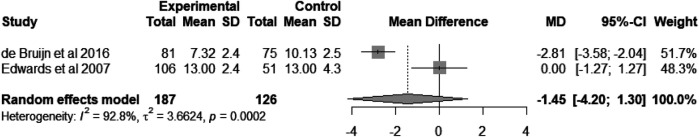
Forest plot showing the mean difference of SF-36(physical component summary) between UAE group and myomectomy or hysterectomy group.

Some studies have used the SF-36 scale to compare the mental and physical health status of patients before and after undergoing UAE vs. myomectomy or hysterectomy. According to reports from two studies involving a total of 313 patients, the pooled mean difference for mental health was −1.58 [95% CI = (-2.29, 0.86), heterogeneity I^2^ = 0.0%, *P* = 0.38; Figure 5], and the pooled mean difference for physical health was −1.45 [95% CI = (-4.20, 1.30), heterogeneity I^2^ = 92.8%, *P* < 0.005; Figure 6] (13, 27). These results indicate that the ability of the UAE group to improve patients' mental health is significantly lower than that of the myomectomy or hysterectomy group. However, no significant difference was observed in physical health outcomes between the two groups, while the substantial heterogeneity (I^2^ = 92.8%) may be attributable to differential effects between myomectomy and hysterectomy surgical approaches.

### Complications

Patients undergoing UAE, myomectomy, and hysterectomy may experience different complications, such as postembolization syndrome, haemorrhage, urinary retention, infection, and vaginal discharge.

Postoperative complications following surgical treatments such as myomectomy or hysterectomy for uterine fibroids can vary depending on the presence or absence of other comorbidities, size and location of the fibroid and the surgical approach such as open or laparoscopic. These complications can be grouped as early (within days to weeks or late (weeks to months) and may range from mild to severe.

### Post-embolization syndrome

**Figure 7:**
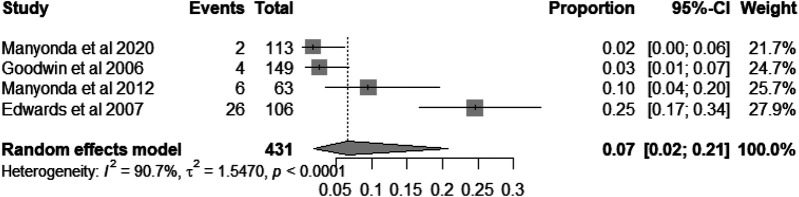
Forest plot showing the incidence rate of postembolization syndrome in patients following UAE.

Postembolization syndrome is a common complication following UAE (8). We analysed the incidence of postembolization syndrome in 431 patients across four studies (8, 18, 20, 27). The pooled incidence rate was 7% [95% CI = (2%, 21%), heterogeneity I^2^ = 90.7%, *P* < 0.005; Figure 7].

### Haemorrhage

**Figure 8:**
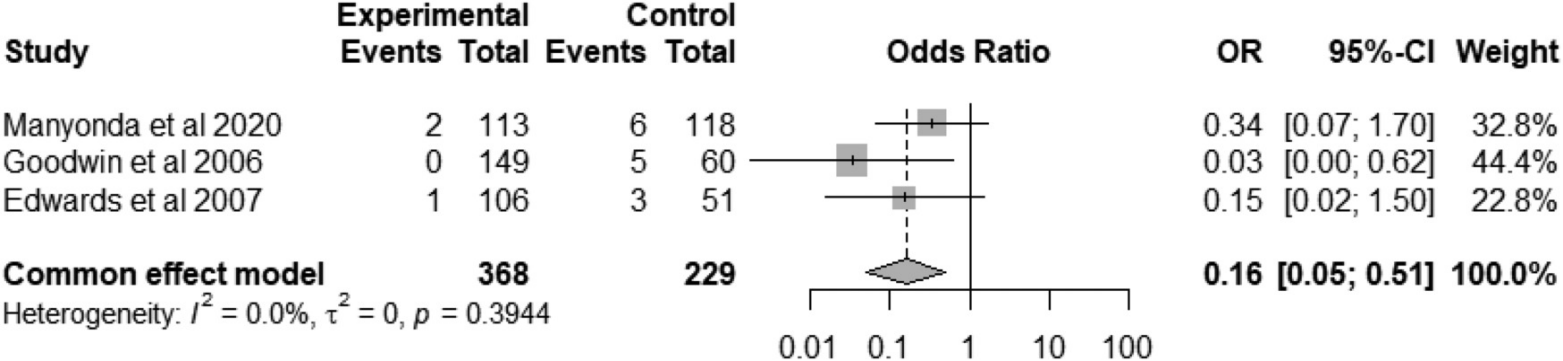
Forest plot showing the incidence of haemorrhage comparisons between the UAE group and myomectomy or hysterectomy group.

Haemorrhage is common following UAE, myomectomy, and hysterectomy. We compared the risk of postoperative bleeding between the UAE group and the group undergoing hysterectomy or myomectomy. The pooled odds ratio (OR) was 0.16 [95% CI = (5%, 51%), heterogeneity I^2^ = 0%, *P* = 0.39; Figure 8], indicating that UAE is 84% less likely to result in haemorrhage compared to hysterectomy or myomectomy (8, 20, 27).

### Urinary retention

**Figure 9:**
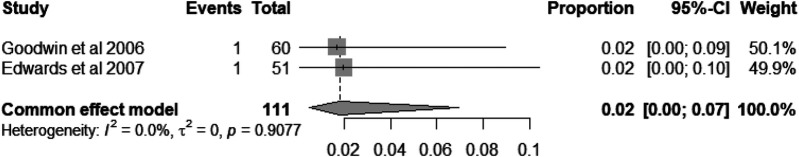
Forest plot showing the incidence rate of urinary retention in patients undergoing myomectomy or hysterectomy.

Urinary retention is a complication following hysterectomy and myomectomy. In two studies involving 111 patients who underwent hysterectomy or myomectomy, the pooled incidence rate of urinary retention was 2% [95% CI = (0%, 7%), heterogeneity *I*^2^ = 0%, *P* = 0.91; Figure 9] (8, 27). While the absence of heterogeneity (*I*^2^ = 0%) and a *P*-value of 0.91 demonstrate consistency between the two studies, the low statistical power stemming from the limited number of included studies may obscure true variability. Additionally, the *P*-value primarily confirms statistical consistency rather than providing clinically meaningful insights.

### Infection

**Figure 10:**
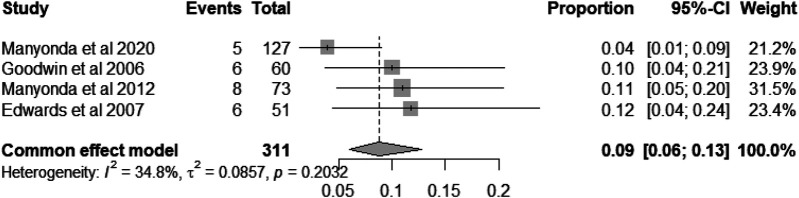
Forest plot showing the incidence rate of infection in patients undergoing myomectomy or hysterectomy.

Patients undergoing hysterectomy and myomectomy may experience infections. Based on reports from four studies involving 311 patients, the pooled incidence rate of infections was 9% [95% CI = (6%, 13%), heterogeneity *I*^2^ = 34.8%, *P* = 0.20; Figure 10] (8, 18, 20, 27).

### Vaginal discharge

**Figure 11:**
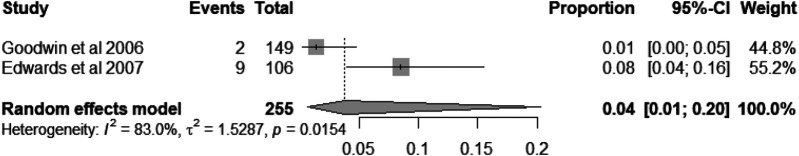
Forest plot showing the incidence rate of vaginal discharge in patients following UAE.

Changes in vaginal discharge are common after UAE. Results from two studies involving 255 patients showed a pooled incidence rate of vaginal discharge of 4% [95% CI = (1%, 20%), heterogeneity *I*^2^ = 83%, *P* = 0.02; Figure 11] (8, 27).

### Publication bias

**Figure 12:**
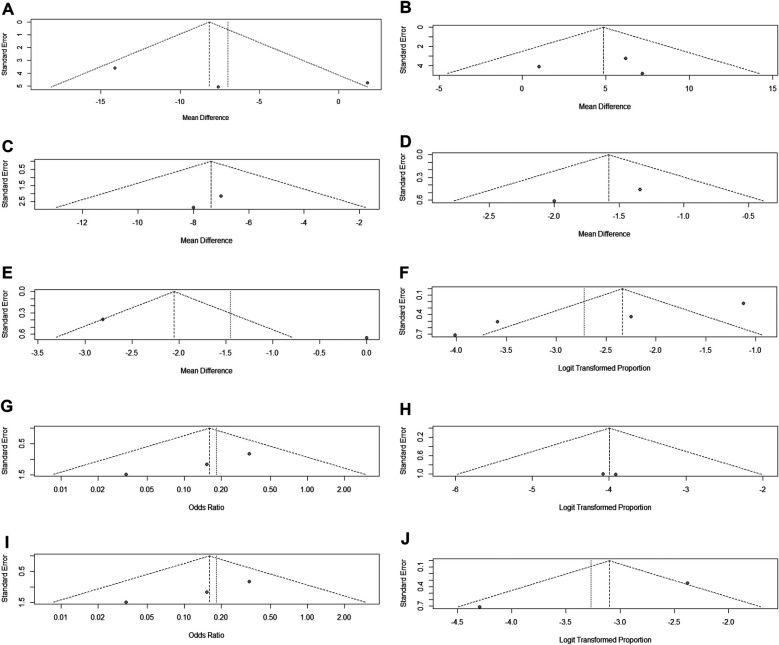
**(A)** Funnel plot for UFS-QoL health-related quality-of-life score. **(B)** Funnel plot for UFS-QoL symptom severity score. **(C)** Funnel plot for EQ-5D-3l score. **(D)** Funnel plot for SF-36(mental component summary) score. **(E)** Funnel plot for SF-36(physical component summary) score. **(F)** Funnel plot for postembolization syndrome. **(G)** Funnel plot for Haemorrhage. **(H)** Funnel plot for urinary retention. **(I)** Funnel plot for infection. **(J)** Funnel plot for vaginal discharge.

No substantial publication bias for UFS-QoL health-related quality-of-life score, UFS-QoL symptom severity score, EQ-5D-3l score, SF-36 (mental component summary), or urinary retention (Figures 12A–D,H). And potential publication bias was observed for SF-36 (physical component summary), postembolization syndrome, haemorrhage, infection, and vaginal discharge (Figures 12E–G,I,J). Notably, the limited number of included studies precludes definitive conclusions regarding whether funnel plot asymmetries originate from publication bias or stochastic variation inherent to small meta-analyses.

## Discussion

The motivation for this systematic review is to provide a comprehensive comparison of the available treatment options for symptomatic uterine fibroids, specifically UAE (8), myomectomy, and hysterectomy, in terms of their impact on women's quality of life, symptom relief, and associated complications. Given the significant impact of uterine fibroids on women's well-being, this review aims to guide clinicians in making informed treatment decisions by evaluating the effectiveness, recovery, and risks of each intervention. The study utilised several tools, including the Uterine Fibroid Symptom and Health-Related Quality of Life Questionnaire (UFS-QoL), European Quality of Life 5 Dimensions 3 Level Version (EQ-5D-3l), and SF-36 scale, to measure health-related QoL and symptom severity before and after the treatments. The principal findings of the study showed no statistically significant difference in the improvement of health-related QoL between UAE and myomectomy, as measured by the UFS-QoL scale. However, UAE was associated with a significant reduction in symptom severity compared to myomectomy. In contrast, when using the EQ-5D-3l scale, both myomectomy and hysterectomy led to significant improvements in QoL compared to UAE. The SF-36 scale revealed that while UAE was less effective in improving mental health, it did not differ significantly from myomectomy or hysterectomy in terms of physical health improvement. Regarding complications, UAE showed a significantly lower risk of haemorrhage, with a reduced likelihood of postembolisation syndrome, but it had a higher incidence of vaginal discharge compared to the other treatments. Myomectomy and hysterectomy were associated with risks of urinary retention and infection, although these complications were less frequent.

The results from this study highlight the need for careful consideration when evaluating and reflecting on current treatment options for UF, as well as the diverse outcomes and complications associated with each approach. Uterine fibroids treatments are linked to a range of complications that vary depending on the intervention type, and these must be carefully balanced against the benefits of symptom relief and treatment efficacy. UAE was found to be associated with adverse events such as menstrual irregularities, menopausal symptoms, vaginal discharge and the potential need for additional interventions to manage persistent symptoms (8, 18, 27, 50). The aetiology is predominantly linked to tissue necrosis resulting from the embolization-induced ischemia, leading to expulsion of necrotic material through the vaginal canal. However, other factors, such as inflammatory responses and secondary infections, have also been implicated in some cases. The variability in reported incidence rates and lack of consensus on standardized diagnostic criteria for pathological discharge post-UAE underscore the need for further high-quality research. Longitudinal studies evaluating the risk factors, preventive strategies, and outcomes of different management approaches are particularly warranted to enhance the evidence base. While UAE provides a minimally invasive option that preserves the uterus, it does not offer a definitive solution, as fibroids may recur or remain symptomatic (26).

Hormonal consequences also contribute to variations in QoL. Hysterectomy accompanied by bilateral oophorectomy induces surgical menopause, characterised by abrupt onset of vasomotor symptoms, mood changes, sleep disturbances, and reduced libido. These symptoms negatively impact QoL domains such as vitality, mental health, and sexual function, particularly in the absence of hormone replacement therapy. In contrast, myomectomy and UAE typically preserve ovarian function, avoiding the systemic effects of sudden hormonal withdrawal.

Recovery profiles and postoperative complications further influence QoL trajectories. Less invasive procedures, such as UAE or laparoscopic myomectomy, are associated with shorter hospital stays, faster return to normal activities, and lower postoperative pain, which can result in earlier improvements in physical and social functioning (51). Conversely, open surgery, such as abdominal hysterectomy or open myomectomy, involves longer recovery periods and a higher risk of complications, potentially leading to delayed or reduced improvements in QoL.

Sociocultural beliefs and psychological perceptions surrounding the uterus and femininity also play a pivotal role. In many cultural settings, the uterus symbolises femininity, fertility, and identity. Removal of the uterus may lead to feelings of loss, diminished self-worth, or anxiety, which can negatively affect emotional and social dimensions of QoL (52). Furthermore, lack of social support, stigma associated with infertility or surgical menopause, and the absence of culturally sensitive counselling may exacerbate these psychological impacts.

Baseline health status and comorbid conditions must be considered. Women with co-existing conditions such as endometriosis, chronic pelvic pain, anaemia, or mental health disorders often report lower baseline QoL and may experience varied degrees of improvement following treatment. These factors act as important confounders and must be accounted for when interpreting QoL outcomes across different intervention groups. However these points are often unreported within research studies.

Hysterectomies, while offering a permanent resolution by completely removing fibroid-affected tissue, comes with a longer recovery period and increased risks related to surgery and anaesthesia. Myomectomy offers the benefit of preserving the uterus, making it a favourable option for women desiring fertility preservation (53). However, it carries its own set of complications, including postoperative pain and the potential for reintervention if fibroids recur, as seen in the results where UAE showed a reduced ability to address severe symptoms in comparison to myomectomy (31, 54). The data indicates that UAE is less likely to result in postoperative haemorrhage compared to hysterectomy or myomectomy. However, the lack of statistical significance and the relatively wide confidence interval highlight the need for caution in drawing definitive conclusions.

Pharmacological treatments, such as GnRH analogues, provide symptom relief and can reduce fibroid size prior to surgery (23, 29). However, their long-term use is associated with menopausal symptoms, including hot flashes, mood swings, vaginal dryness, and an increased risk of osteoporosis. These medications induce a hypoestrogenic or hypoandrogenic state, which may negatively affect a woman's quality of life (34, 55). To mitigate these effects, hormone replacement therapy (56) is often prescribed in conjunction with GnRH analogues, but this introduces additional considerations and potential risks. Long-term use of GnRH analogues requires careful monitoring due to risks such as bone density reduction and cardiovascular issues. The prohibitive cost of these therapies presents a significant barrier in low- and middle-income countries (LMICs), where financial constraints may limit access to treatment. This disparity highlights the inequities in global healthcare, as patients in LMICs may be unable to afford these medications, leading to missed opportunities for symptom management and treatment.

Preventing fibroids recurrence remains challenging due to the genetic and hormonal influences on fibroid growth. While complete prevention is not possible, several strategies may help reduce recurrence. Lifestyle and dietary adjustments, such as consuming diets rich in fruits, vegetables, and whole grains, may support hormonal balance and reduce the risk of recurrence. Avoiding high-fat, processed foods, especially red meats, is recommended due to their association with increased fibroid risk. Regular physical activity can help with weight management, potentially contributing to hormonal equilibrium. Additionally, hormonal therapies like low-dose oral contraceptives or GnRH analogues can be used to manage oestrogen levels post-treatment, but their use requires careful monitoring due to potential side effects (57). Emerging treatments such as selective progesterone receptor modulators like ulipristal acetate and supplements like vitamin D or anti-inflammatory agents, such as curcumin, show promise, though further research is needed to confirm their effectiveness (47).

### Clinical implications

This review has a number of clinical implications. First, the findings of this systematic review shows the importance of considering treatment options based on individual patient factors. For women with severe symptoms of uterine fibroids who do not respond to medical therapy, UAE offers a less invasive approach with fewer risks of haemorrhage and quicker recovery. However, myomectomy and hysterectomy may offer better overall improvements in quality of life, especially mental health, and are more effective in reducing symptoms in the long term. Second, healthcare providers should discuss the potential benefits and risks of each treatment option with patients, particularly considering the long-term implications for mental health and symptom management. Thirdly, clinicians should be aware of specific complications linked to each treatment, such as postembolisation syndrome following UAE, and monitor patients accordingly.

### Research implications

Regarding research implications of the present review, the analysis indicated a potential publication bias in studies on postembolisation syndrome, suggesting that more research is needed to explore this complication and its long-term effects. The study emphasises the need for further research focusing on long-term outcomes of each treatment modality for uterine fibroids, particularly in terms of sustained symptom relief and QoL improvements over several years. Additionally, the variation in tools used to assess QoL (UFS-QoL, EQ-5D-3l, SF-36) calls for more standardised metrics to improve comparability between studies and provide clearer clinical guidelines.

### Strengths and limitations

The study sample primarily consisted of research from high-income countries (HICs), which limits the applicability of the findings to women in LMICs, where healthcare access and treatment options are more limited. This imbalance in global health research reflects systemic inequities in healthcare funding, infrastructure, and access to innovative treatments, which disproportionately impact women's health in LMICs. Patients in these regions may rely on alternative or more invasive treatments due to a lack of access to advanced medical interventions, further exacerbating health disparities. This gap in research also underscores the need for more region-specific studies to develop culturally sensitive and effective treatments tailored to the unique needs of women in LMICs.

Although a total of 42 studies were initially screened, only 5 met the predefined inclusion criteria for the meta-analysis. This limited number reflects the rigorous selection process adopted to ensure methodological quality, data consistency, and relevance to the research objective. While we acknowledge that the small number of included studies may affect the generalisability and statistical power of the findings, the selected studies were of high methodological quality and provided sufficient data for meaningful synthesis. This limitation highlights the need for more robust and standardized research in this area to strengthen the evidence base for future meta-analyses.

The precision of certain estimates—particularly complications like postembolisation syndrome incidence—is limited by both the small number of included studies and low event counts, resulting in wide confidence intervals that necessitate cautious interpretation. Small-study effects analyses (Egger's test & funnel plots) exhibit diminished sensitivity when few studies are available. With <10 studies in key comparisons, both Type II error risk in statistical tests and subjective interpretation of graphical asymmetry necessitate cautious interpretation of publication bias evaluation.

A notable limitation of this meta-analysis is the lack of consistent reporting on race and ethnicity across the included studies. This gap is particularly significant in the context of uterine fibroids, as numerous studies have shown that fibroid prevalence, symptom severity, and response to treatment vary markedly across different ethnic groups, with women of African descent experiencing a disproportionately higher burden. The absence of stratified race/ethnicity data limits our ability to assess differential treatment outcomes and may mask clinically relevant disparities. Consequently, the generalisability of our findings to diverse populations is constrained. Future research should prioritise the inclusion and transparent reporting of race and ethnicity data, as well as perform subgroup analyses, to support more equitable and evidence-based care for all patients affected by fibroids.

## Conclusion

Although UAE, myomectomy, and hysterectomy each have their advantages and risks, myomectomy and hysterectomy tend to offer better long-term improvements in quality of life. However, UAE can be a good alternative for women seeking less invasive options with fewer risks of haemorrhage. Clinicians should offer a personalised treatment plan, considering patient preferences, symptom severity, and the potential risks and benefits of each procedure. There is a need for further large-scale studies to compare the long-term outcomes of these treatments, including the effects on fertility, recurrence of fibroids, and mental health. Research should also focus on minimising complications, particularly postembolisation syndrome and vaginal discharge in UAE patients. The lack of representation in global fibroids research from LMICs limits healthcare professionals’ ability to make evidence-based decisions and implement effective interventions. It is crucial to foster global collaboration in research to ensure equitable representation and address the unmet needs of women worldwide. Such efforts can help bridge the gap between developed and developing nations, ensuring that all women, regardless of their geographic location, have access to the most effective and appropriate treatments for uterine fibroids. By prioritising these areas, healthcare systems can improve outcomes and ensure that women across all income settings have access to the care they need.

## Data Availability

The original contributions presented in the study are included in the article/Supplementary Material, further inquiries can be directed to the corresponding author.
